# Comparison of the empirical linear ablation and low voltage area-guided ablation in addition to pulmonary vein isolation in patients with persistent atrial fibrillation: a propensity score-matched analysis

**DOI:** 10.1186/s12872-022-02460-9

**Published:** 2022-01-22

**Authors:** Noriyuki Suzuki, Shinji Kaneko, Masaya Fujita, Masanori Shinoda, Ryuji Kubota, Taiki Ohashi, Yosuke Tatami, Junya Suzuki, Hitomi Hori, Kentaro Adachi, Ryota Ito, Yoshinori Shirai, Satoshi Yanagisawa, Yasuya Inden, Toyoaki Murohara

**Affiliations:** 1 Department of Cardiology, JA Aichi-Koseiren Toyota Kosei Hospital, Toyota, Aichi Japan; 2 Department of Cardiology, Nagoya University Graduate School of Medicine, 65 Tsurumai, Showa, Nagoya, Aichi 466-8550 Japan

**Keywords:** Catheter ablation, Linear ablation, Pulmonary vein isolation, Persistent atrial fibrillation, Low voltage area

## Abstract

**Background:**

The efficacy of pulmonary vein isolation (PVI) alone is not guaranteed for persistent atrial fibrillation (PeAF), and it is unclear which type of ablation approach should be applied in addition to PVI. This study aimed to compare outcomes and prognosis between empirical linear ablation and low-voltage area (LVA) ablation after PVI for PeAF.

**Methods:**

We enrolled 128 patients with PeAF who were assigned to the linear ablation group (n = 64) and the LVA ablation group (n = 64) using a propensity score-matched model. After PVI and cardioversion, the patients underwent either empirical linear ablation or LVA ablation during sinus rhythm. All patients in the linear ablation group underwent both roof line and mitral valve isthmus (MVI) ablations. An electrical-guided ablation targeting LVA (< 0.5 mV) was performed in the LVA group. When there was no LVA in the LVA group, only PVI was applied. We compared the procedural outcomes and recurrence after ablation between the two groups.

**Results:**

The baseline characteristics were well-balanced between the two groups. Fifty patients had LVA (22 and 28 patients in the linear and LVA groups). The roof and MVI lines were completed in 100% and 96.9% of the patients. During the mean follow-up of 279.5 ± 161.3 days, the LVA group had significantly lower recurrence than the linear group (15 patients [23%] vs. 29 patients [45%], *p* = 0.014). Thirty-five patients were prescribed antiarrhythmic drugs during the follow-up period (linear group, n = 17; LVA group, n = 18); amiodarone and bepridil were administered to most of the patients (15 and 17 patients, respectively). The difference in the prognosis was relevant among the patients with LVA, while this trend was not observed in those without LVA. The LVA ablation group demonstrated significantly lower radiofrequency energy and shorter procedural time compared to the linear ablation group. The recurrence of atrial flutter was more likely to occur in the linear group than in the LVA group (14 [22%] vs. 6 [9.4%], *p* = 0.052).

**Conclusion:**

The electrophysiological-guided LVA ablation is more effective than empirical linear ablation in PeAF patients with LVA. Unnecessary empirical linear ablation might have a risk of iatrogenic gap and atrial flutter recurrence.

**Supplementary Information:**

The online version contains supplementary material available at 10.1186/s12872-022-02460-9.

## Background

Catheter ablation for atrial fibrillation (AF) is an effective treatment for maintaining normal sinus rhythm. Pulmonary vein isolation (PVI) is a promising method of ablation for patients with paroxysmal AF [1, 2], but the success rates for persistent AF (PeAF) have not paralleled those for paroxysmal AF [3].

Adjunctive ablations, such as ablation of complex fractionated atrial electrogram (CFAE), ganglionated plexi, linear lesion ablation, and low voltage area (LVA) ablation, have been proposed to improve procedural efficacy [4–6]. Among them, linear ablation attempts to modify the left atrium (LA) as a conventional approach with proven efficacy [7]. However, creating complete (and durable) conduction block along a linear lesion is sometimes challenging [8]. In particular, ablation of the mitral valve isthmus (MVI) was achieved in 75% of patients in the Star AF II study, a large-scale randomized study [9], and the authors concluded that there was no reduction in AF recurrence when either linear ablation or CFAE ablation was performed in addition to PVI. Moreover, it is unclear whether completed linear ablation as a substrate modification of LA improves outcomes, especially in patients without documented atrial flutter (AFL) during the procedure. In contrast, several studies have reported that an additional LVA substrate modification targeting the damaged and arrhythmogenic atrial tissue is effective in improving the outcomes [10–12]; however, LVA ablation has not yet become the cornerstone of the PeAF ablation strategy. It is unclear which type of ablation strategy should be applied in patients with PeAF in addition to PVI in clinical practice [4–6].

Therefore, the present study was conducted to compare the outcomes and prognosis between empirical linear ablation and electrical-guided LVA ablation, which are commonly used as standard approaches in patients undergoing catheter ablation for PeAF.

## Methods

### Patient population

We initially assessed 144 patients with PeAF who underwent catheter ablation for AF at Toyota Kosei Hospital between November 2016 and June 2018 retrospectively. The indications for catheter ablation were as per the most recent guidelines [13, 14].

**Fig. 1:**
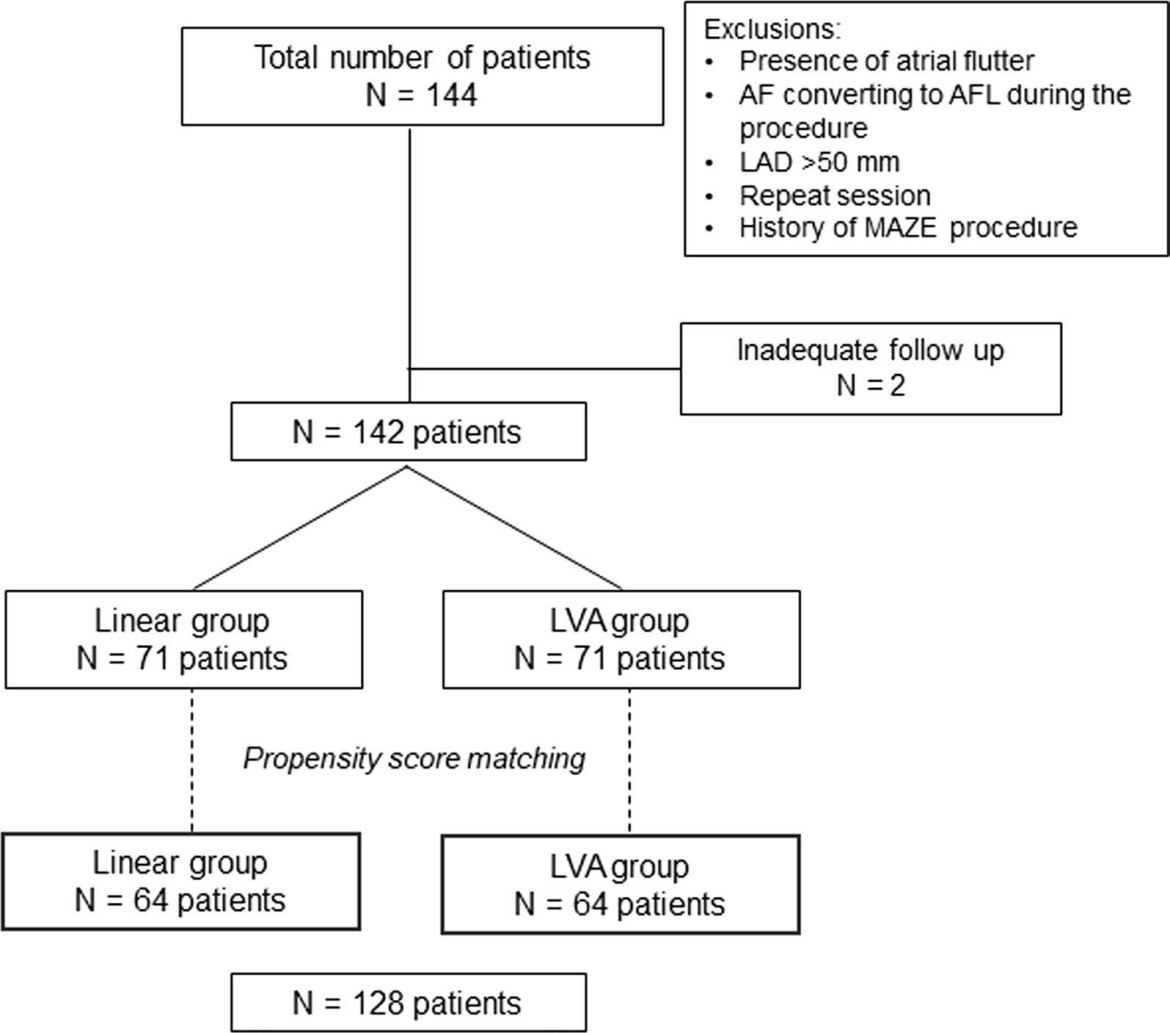
Flowchart of the study. *AF* atrial fibrillation, *AFL* atrial flutter, *LAD* left atrial diameter, *LVA* low-voltage area

All patients were alternatively assigned to the linear ablation group (linear group) or the LVA ablation group (LVA group) after PVI (Fig. 1). We excluded patients with the following: inadequate follow-up after the procedure; the presence of AFL and AF conversion to AFL requiring specific linear ablation during the procedure; a LA diameter > 50 mm; repeat session; and history of MAZE procedure. All patients underwent cardioversion after PVI, and an LA voltage map was generated during sinus rhythm unless AF terminated the sinus rhythm during PVI. Patients in the linear ablation group underwent empirical linear ablation, while electrical-guided ablation targeting LVA was applied in the LVA group. Although we generally applied each ablation approach alternatively so that the number of patients in each group was the same, there were some cases in which the operators decided to adopt the ablation approach preferentially based on the patients’ characteristics in a non-randomized manner. However, the decision of which ablation approach would be assigned had been made before the voltage map creation following PVI, and therefore, if there was no LVA in the LVA ablation group, we performed PVI ablation only. We did not control for the number of patients with LVA and those with LVA in each ablation group.

In the crude population, 71 patients were assigned equally to each group (142 patients in total). However, different baseline characteristics and examination data were observed between the two approach groups in the non-randomized study design (Additional file 1: Table S1). We subsequently constructed a propensity score model for the linear or LVA group to minimize differences and overcome any bias in the baseline characteristics due to the study design. A total of 128 matched patients (linear and LVA, 1:1) were included in the analysis. Informed consent was obtained from all patients prior to the procedure. The study protocol was approved by the Institutional Review Board of the study hospital. The study was performed in compliance with the principles of the Declaration of Helsinki.

PeAF was defined as AF lasting for > 7 days, and the study also enrolled patients with longstanding AF lasting for > 1 year [13, 14]. All patients with PeAF underwent cardiac computed tomography (64-slice) and transthoracic echocardiography before ablation to evaluate the presence of thrombi and the LA volume. Transesophageal echocardiography was not routinely performed before procedure, but all patients underwent the contrast-enhanced computed tomography for the exclusion of possibility of intracardiac thrombi. All antiarrhythmic drugs were suspended before the ablation procedure; specifically, amiodarone and bepridil were suspended for ≥ 1 month.

### Ablation procedures

Patients continued anticoagulant therapy for at least 3 weeks before the ablation procedure. Anticoagulant drugs including vitamin K antagonists and direct oral anticoagulants were uninterruptedly administered throughout the procedure [15].

The procedure was performed under mild intravenous sedation and analgesia. Before transseptal puncture, patients received intravenous unfractionated heparin and maintained an activated clotting time of > 300 s. Following transseptal puncture, the LA geometry was evaluated using a three-dimensional electroanatomical mapping system (EnSiteNavx, St. Jude Medical, St. Paul, MN, USA). The radiofrequency (RF) ablation was performed with settings of 25–40 W and a temperature limit of 40 °C using an irrigated ablation catheter (the TactiCath™ Quartz contact force sensing or FlexAbility™ irrigated ablation catheter; St. Jude Medical). We used a Swartz sheath (St. Jude Medical) for LA ablation, but did not use a steerable sheath. A point-by-point PVI was performed with the target contact force sensing > 10 g, lesion index > 4–5, and lesion distance of 4 mm, if necessary [16, 17]. The lesion index was monitored during the PVI. Cardioversion was performed in cases where AF persisted after PVI. Bi-directional PVI was confirmed by pacing inside the pulmonary vein (PV) and documentation of an exit block out of the PV, and vice versa. All patients underwent cavotricuspid isthmus (CTI) ablation. Superior vena cava isolation was not performed in this study population.

Voltage mapping in the LA was generated during sinus rhythm in all patients. The LVA was defined as an area with a bipolar peak‐to‐peak voltage amplitude < 0.5 mV [18, 19], as measured by a 20-pole multipolar circular ring catheter with 1–2.5–1 mm electrode spacing (Inquiry™ Afocus™II, Abbott). More than 500 points in the LA were acquired to create a voltage map in each case. We defined patients with LVA ≥ 5 cm^2^ in the LA as those with LVA [18]. We did not routinely perform post-ablation induction maneuvers of pacing stimulation and isoproterenol infusion in this study.

### Linear ablation

**Fig. 2:**
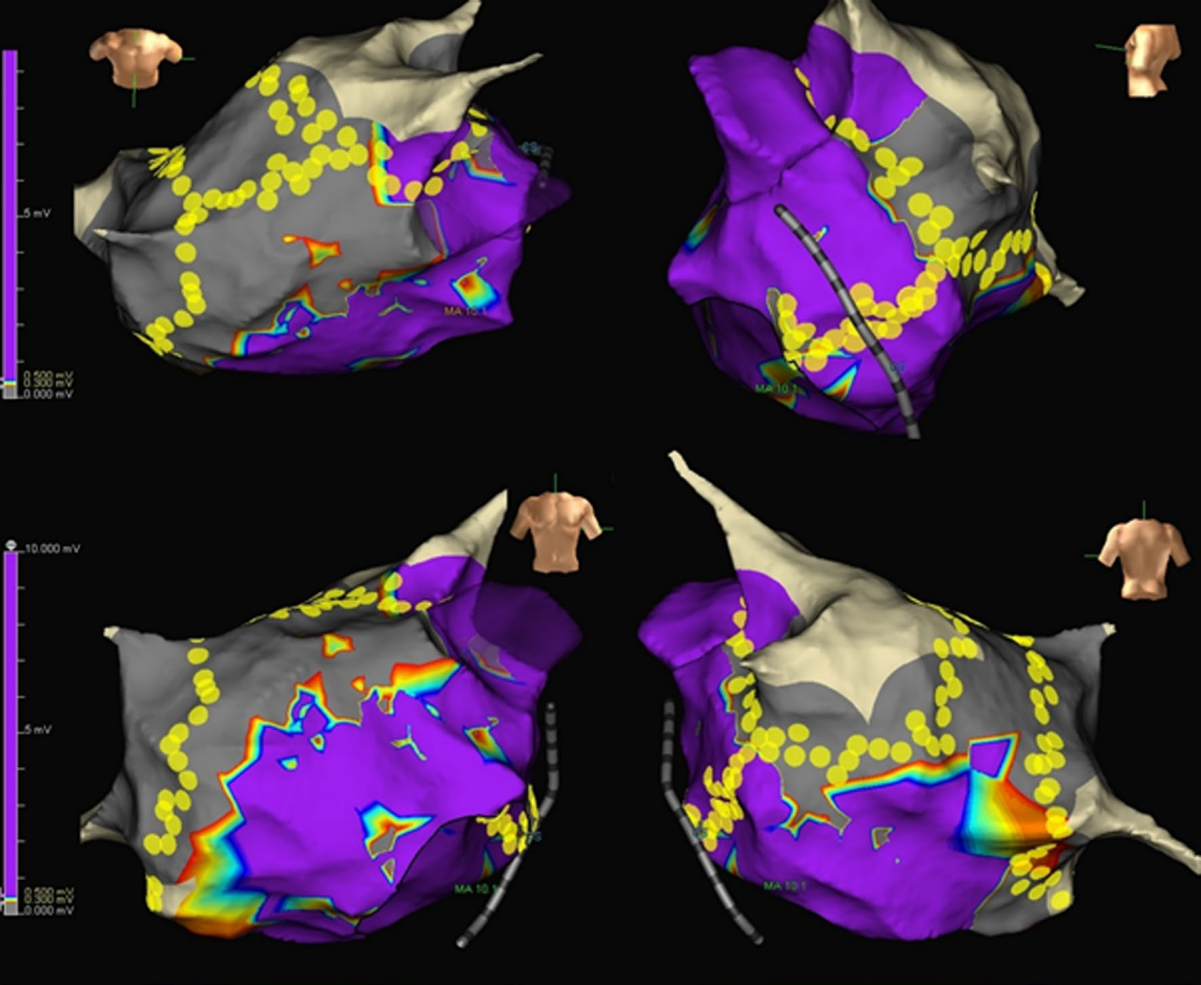
Representative case of PVI and linear ablation sites. Linear ablation, inclusive of the roof line and MVI, was performed. Roof-line ablation was performed for the left atrium between the contralateral superior PVs, and MVI line ablation was performed from the 4 or 5 o’clock position on the mitral annulus and up to the 2 o’clock position on the ostium of the left inferior PV. The yellow tags indicate ablation points of PVI, roof line, and MVI line. In the voltage map, dark purple and gray colors represent voltage amplitudes of 0.5 mV and 0.3 mV, respectively. The purple color represents the healthy area with an electrogram amplitude of ≥ 0.5 mV. *PVI* pulmonary vein isolation, *MVI* mitral valve isthmus, *PV* pulmonary vein

In the linear ablation group, both roof line and MVI linear ablation were performed during sinus rhythm (Fig. 2). Roof-line ablation was applied for the LA between both contralateral superior PVs, and MVI line ablation was performed from the 4 or 5 o’ clock position on the mitral annulus and up to the 2 o’clock position on the ostium of the left inferior PV. We did not create an anterior mitral isthmus line as a first-line choice because the strategy of the first-line lateral MVI ablation has been adopted for a long time in our institution [20]. If it was difficult to completely ablate the MVI line from the endocardial side, we ablated the epicardial side over the coronary sinus (CS) with a maximum output of 30 W, being careful to prevent steam pop formation. After CS ablation, we confirmed no stenosis of the CS by injection of contrast agent through the CS electrode catheter. When the MVI was still not completed after the above-mentioned approaches, we attempted further endocardial ablation with an increased output energy of 35–40 W on the mitral annulus energetically, where the electrical potential remained. Even at this stage, we did not apply an additional anterior or anterior lateral line to compensate for the block line. The MVI block was confirmed using the differential pacing technique of the endocardial sides and CS. In all cases, only roof and MVI line ablations were performed after PVI, without an additional trigger or linear ablation.

### LVA ablation

**Fig. 3:**
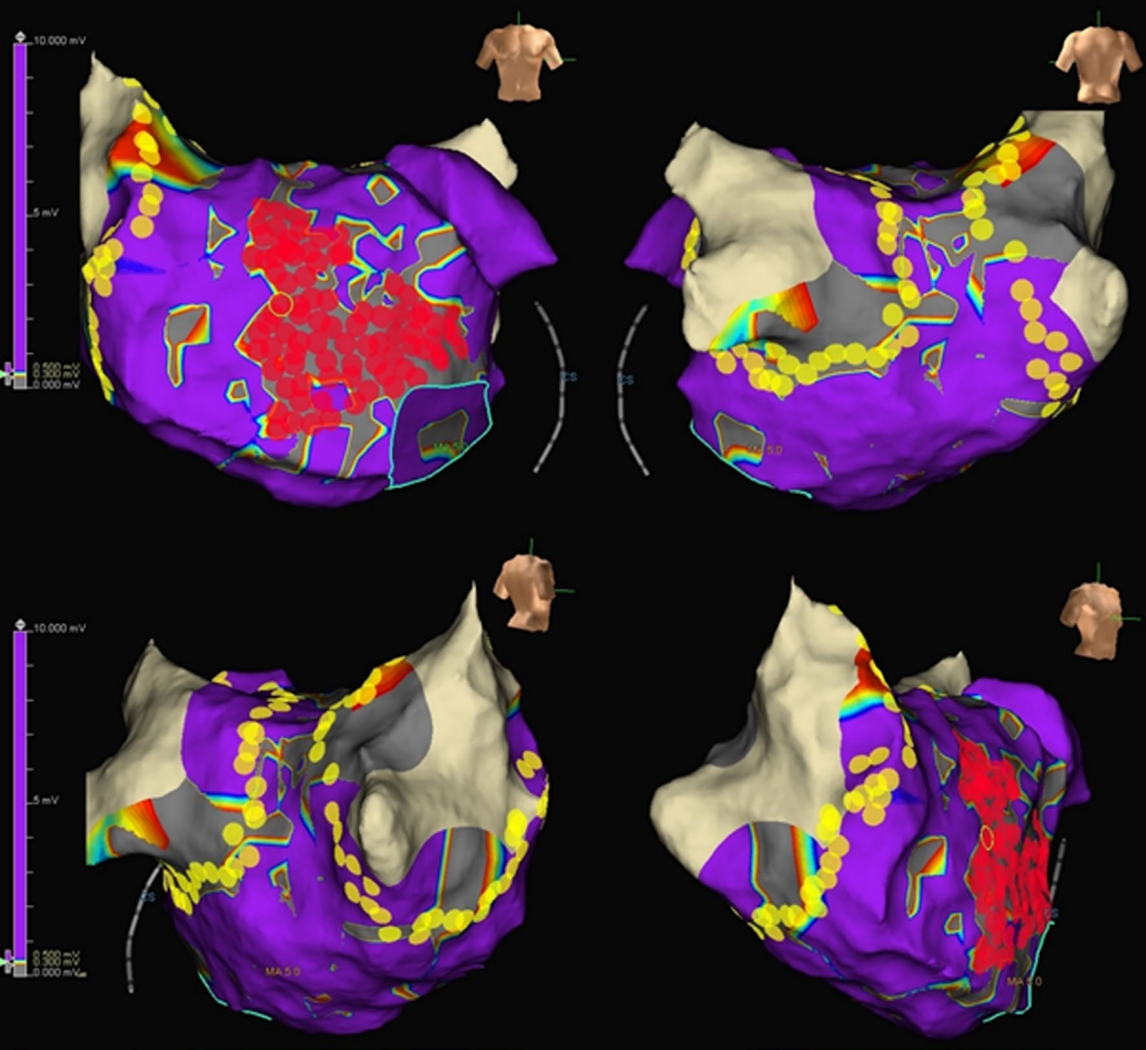
Representative case of PVI and LVA ablation. Voltage mapping was performed during sinus rhythm, and LVA was defined as an area with a bipolar voltage amplitude < 0.5 mV. In the voltage map, dark purple and gray colors represent voltage amplitudes of 0.5 mV and 0.3 mV, respectively. The purple color represents the healthy area with an electrogram amplitude of ≥ 0.5 mV. In this case, large LVAs were identified on the anterior wall of the left atrium, which was a target for the ablation. The areas inside the LVA were ablated for homogenization and until loss of pacing capture was achieved (red tags). *PVI* pulmonary vein isolation, *LVA* low voltage area

For the LVA ablation group, we ablated areas inside the LVA of the voltage amplitude < 0.5 mV, which were closely aggregated in the LA for homogenization (Fig. 3). A general power setting of 25–40 W with target contact force sensing of > 10 g, lesion index of > 4.0, and lesion distance of 4 mm were applied during LVA ablation. If there was no LVA, no further ablation was performed. The endpoint of LVA ablation was defined as the absence of local electrical potential in the ablation catheter and failure to capture the local myocardium inside the LVA (pacing output: 9.9 V). LVA ablation was performed in the LA only, but not in the right atrium.

### Follow-up

All patients received anticoagulant drugs for at least 3 months after the procedure. Transthoracic echocardiography was performed 3 months after discharge. All patients were regularly followed up at 3, 6, 9, and 12 months after ablation in outpatient clinic of our institution. At each follow-up visit, patients underwent electrocardiogram and were asked about any symptoms related to the presence of arrhythmia. The 24-h Holter monitoring was performed at 3, 6, 9, and 12 months after ablation. Additional surface 12-lead electrocardiogram was scheduled with a short follow-up duration when patients reported palpitations with a suspicion of the recurrence, if necessary. Acute phase AF recurrence within < 3 months of blanking period was not considered a true recurrence. When the patients had early recurrence or frequent supra premature atrial contractions within the blanking period, the antiarrhythmic drugs that were suspended before the procedure were administered again, and we usually continued these medications beyond the blanking period thereafter. AF recurrence was defined as AF/AFL lasting > 30 s on the examination testing after a 3-month blanking period regardless of the administration of antiarrhythmic medication.

### Second session

Patients with AF recurrence were encouraged to undergo repeat sessions when the patients suffered from repetitive AF attacks following antiarrhythmic drug administration. During the second session, we checked the PV reconnection and linear block lines that were created in the previous session (CTI, MVI, and roof line) and compared each site of recurrence between the two groups. For cases complicated with AFL, an activation map was created using a three-dimensional electroanatomical mapping system (EnSiteNavx) to identify the AFL circuit. The diagnosis of Marshall-related AFL was made by missing isochrones through the endocardial map along the MVI line and sudden emerging electrical activation as a focal pattern at the LA roof-LA appendage, PV ridge, and proximal posterior side of the CS, where the Marshall bundle is typically located close to the LA endocardial site. The same post-pacing interval to cycle length of the target AFL and successful termination of the AFL through ablation on these endocardial sites could support the possible involvement of the Marshall bundle in the AFL. We did not routinely insert a micro-electrical catheter into the Marshall vein for diagnosis in the study population.

### Study endpoints

The primary endpoint was the comparison of AF recurrence between the linear and LVA groups after the blanking period regardless of the administration of antiarrhythmic medication. The secondary endpoints were differences in procedural time, fluoroscopy time and dose between the two groups. We additionally evaluated the prognoses of the recurrence rate after ablation between the two ablation approaches in patients with and without LVA.

### Statistical analysis

The patients’ characteristics and procedural data are expressed as mean ± standard deviation or median (first and third quartiles). Differences in baseline characteristics were analyzed using Student’s *t*-test for parametric data and the Mann–Whitney *U* test for non-parametric data. Categorical variables were compared using the chi-square test or Fisher’s exact test. Survival curves were generated using Kaplan–Meier estimates, and time-to-event analyses were performed using the log-rank test. For propensity score-matched analysis, we calculated the propensity score using a multivariable logistic regression model using linear ablation as the dependent variable and including the following baseline factors: age, male sex, duration of AF, LA diameter, left ventricular ejection fraction, and CHADS2 and CHA2DS2-VASc scores. Thereafter, 1:1 nearest-neighbor greedy matching was performed. All statistical analyses were performed using SPSS statistical ver. 23.0. The significance level was set at *p* < 0.05.

## Results

### Patients’ characteristics

The baseline characteristics between the linear group (n = 64) and LVA group (n = 64) after propensity score matching analysis are shown in Table 1. The parameters were well-balanced with no significant differences in age, sex, CHADS2 and CHA2DS2-VASc scores, AF duration, LA diameter, left ventricular ejection fraction, and LA volume between the linear and LVA groups.

**Table 1:** Comparison of baseline characteristics between the linear and LVA groups after propensity score matching analysis

	Linear group (n = 64)	LVA group (n = 64)	*p* value
Age (years)	66.4 ± 10.9	70.0 ± 11.3	0.103
Men (%)	49 (76.6)	48 (75.0)	0.837
CHADS2 score	1.8 ± 1.2	1.97 ± 1.4	0.453
CHA2DS2-VASc score	2.7 ± 1.6	3.1 ± 1.8	0.254
AF duration (months)	4.5 (3.0–12.8)	8.0 (3.0–16.8)	0.479
Long-standing persistent AF (> 1 year) (%)	23 (35.9)	26 (40.6)	0.585
LAD (mm)	45.4 ± 7.0	46.0 ± 5.6	0.585
LVEF (%)	59.6 ± 14.4	59.2 ± 14.4	0.894
LA volume (mL)	77.3 ± 26.7	83.0 ± 25.1	0.226
Antiarrhythmic drugs (n)	15 (23.4)	16 (25.0)	0.838
Class I	2 (3.1)	1 (1.5)	
Amiodarone or Bepridil	13 (20.3)	15 (23.4)	
DOACs (n)	62 (96.8)	63 (98.4)	0.563
VKA (n)	2 (3.1)	1 (1.5)	0.563

*LVEF* left ventricular ejection fraction, *LAD* left atrial dimension, *AF* atrial fibrillation, *LA* left atrium, *LVA* low voltage area, *DOACs* direct oral anticoagulants, *VKA* vitamin K antagonists. Data are presented as mean ± standard deviation, median (first and third quartiles), or number (percentage)

### Ablation procedural data

The ablation procedural data are presented in Table 2. PVI and CTI ablations were successfully achieved in all patients. No major procedural complications, such as pericardial tamponade, stroke, or embolic events, occurred. Twenty-two patients (34.3%) in the linear group and 28 patients (43.8%) in the LVA group had LVA in the LA (*p* = 0.365). The extent of LVA was not significantly different between the linear group and LVA group (7.12 ± 18.0 cm^2^ and 10.1 ± 16.2 cm^2^; *p* = 0.332). The LVA did not differ significantly within the patients with LVA between the two approach groups (22.3 ± 18.5 cm^2^ and 19.7 ± 26.7 cm^2^; *p* = 0.686). In the linear ablation group, complete bidirectional block of the roof line and MVI line was achieved in 64 (100%) and 62 (96.9%) patients, respectively; in 2 patients, the completed MVI block line could not be achieved despite repetitive ablation. One patient in the LVA group had an extensive LVA in the LA anterior wall, which increased the risk of electrical isolation of the LA appendage; as a result, we made a slight modification to the LVA for this patient. The linear group had a significantly longer procedural time (2.6 ± 0.6 vs. 2.3 ± 0.6 h; *p* < 0.001) and amount of RF energy (103,972 ± 33,190 vs. 81,318 ± 27,200 J; *p* < 0.001) compared to the LVA group. In contrast, there was no significant difference between the groups in the fluoroscopy time (55.6 ± 20.8 vs. 53.4 ± 56.0 min; *p* = 0.771) or fluoroscopy dose (399.2 ± 596.0 vs. 487.4 ± 1,586.1 mGy; *p* = 0.684).

**Table 2:** Comparison of procedure results between the linear and LVA groups after propensity score matching analysis

	Linear group (n = 64)	LVA group (n = 64)	*p* value
Pulmonary vein isolation	64 (100)	64 (100)	n/a
CTI block line	64 (100)	64 (100)	n/a
Roof line	64 (100)	n/a	
MVI line	62 (96.9)	n/a	
LVA (cm^2^)	7.1 ± 18.0	10.1 ± 16.2	0.322
Patients with LVA (%)	22 (34.4)	28 (43.8)	0.365
LA area (cm^2^)	121.9 ± 32.1	113.6 ± 41.1	0.224
LVA/LA (%)	6.6 ± 1.6	10.4 ± 2.5	0.345
Contact force-sensing catheter			
RF (J)	103,972 ± 33,190	81,318 ± 27,200	< 0.001
Procedural time (h)	2.6 ± 0.6	2.3 ± 0.6	< 0.001
Fluoroscopy time (min)	55.6 ± 20.8	53.4 ± 56.0	0.771
Fluoroscopy dose (mGy)	399.2 ± 596.0	487.4 ± 1586.1	0.684
Major complications (%)	0 (0)	0 (0)	n/a

*CTI* cavotricuspid isthmus, *LA* left atrium, *LVA* low voltage area, *MVI* mitral valve isthmus, *RF* radiofrequency. Data are presented as mean ± standard deviation or number (percentage)

### AF recurrence

**Fig. 4:**
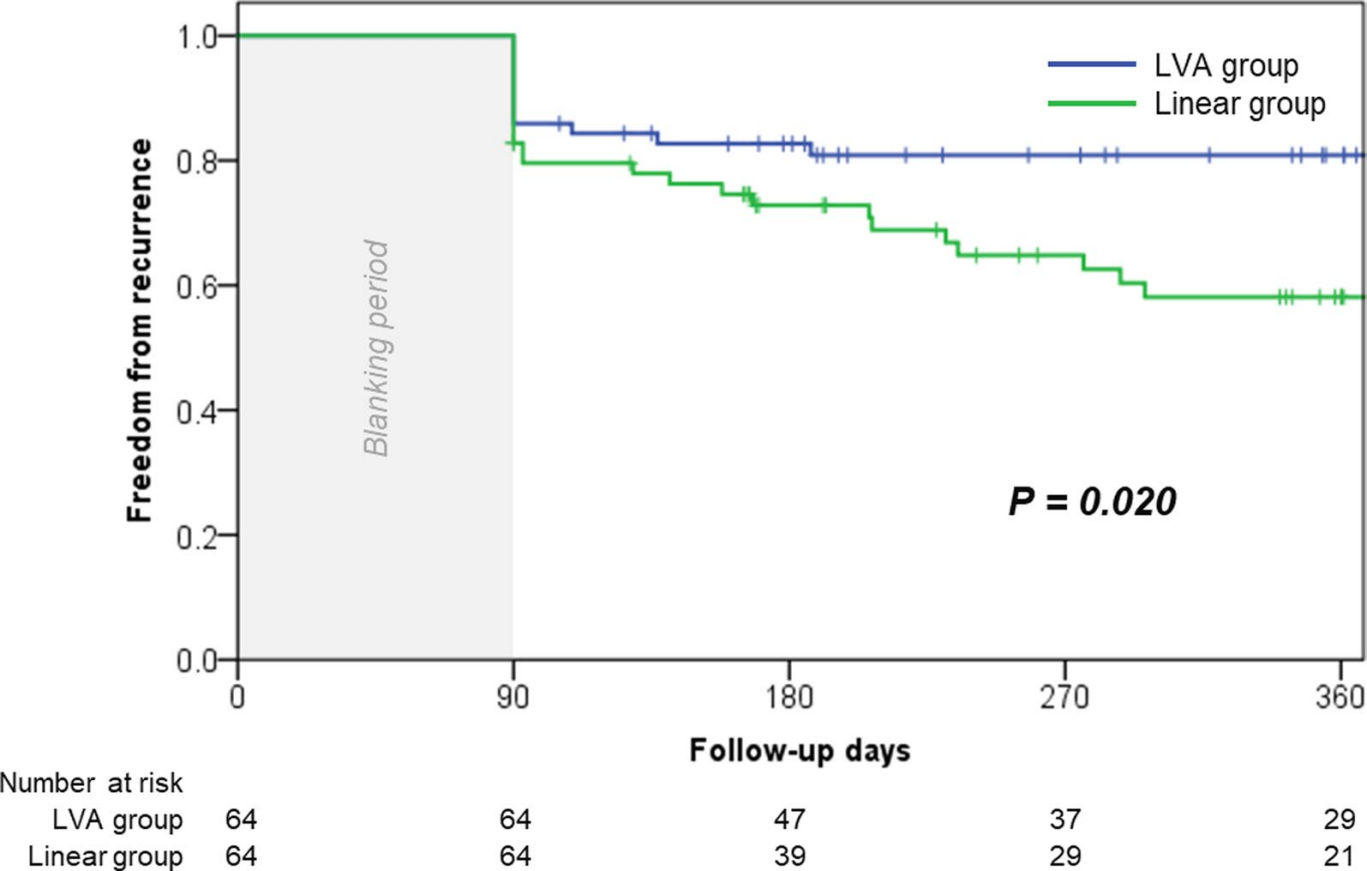
Kaplan–Meier event-free survival curves of recurrence-free rate after ablation between the linear and LVA groups. *LVA* low voltage area

During the mean follow-up of 279.5 ± 161.3 days (from the ablation day to first recurrence or last day of visit), 29 (45%) and 15 (23%) patients in the linear and LVA ablation groups had recurrence, respectively (*p* = 0.014) (Table 3). Kaplan–Meier survival curve analysis demonstrated that the LVA group exhibited a significantly better prognosis for recurrence-free AF than the linear group (log-rank *p* = 0.020; Fig. 4). Thirty-five patients were prescribed antiarrhythmic drugs after ablation beyond the blanking period during follow-up (linear group, n = 17; LVA group, n = 18). Amiodarone and bepridil were administered to the majority of patients (15 and 17 patients, respectively). No adverse events were caused by the administration of amiodarone and bepridil during the follow-up period. As for the recurrence rhythm type, AFL recurrence was more likely to occur in the linear group than in the LVA group (14 [22%] vs. 6 [9.4%], *p* = 0.052).

**Table 3:** Clinical outcomes and prognoses

	Linear group (n = 64)	LVA group (n = 64)	*p* value
Recurrence (%)	29 (45.3)	15 (23.4)	0.009
Early recurrence (%)	13 (20.3)	9 (14.1)	0.353
Recurrence type			
AF (%)	13 (20.3)	7 (10.9)	0.146
AFL (%)	14 (21.9)	6 (9.4)	0.052
Second session (%)	20/29 (31.4)	11/15 (17.2)	0.763
Recurrence at PV	14/20 (70.0)	6/11 (54.4)	0.390
Recurrence at CTI line	0/20 (0)	0/11 (0)	n/a
Recurrence at MVI	5/20 (25.0)	n/a	n/a
Recurrence at roof line	8/20 (40.0)	n/a	n/a
Marshall AFL	4/20 (20.0)	0/11 (0)	0.269

*AF* atrial fibrillation, *AFL* atrial flutter, *PV* pulmonary vein, *MVI* mitral valve isthmus, *LVA* low voltage area. Data are presented as number (percentage)

### Second ablation session

Thirty-one patients (20 patients and 11 in the linear and LVA groups, respectively) underwent a second session of AF ablation due to recurrence, and PV reconnection was observed in 14 patients and 6 patients, respectively. In the linear group, recurrence of the MVI line was observed in 25% of patients (5/20), while roof line reconnection was found in 40% (8/20) of the patients, as confirmed by intracardiac electrograms. No recurrence was observed at the CTI line in either group. Additionally, AFL through the vein of Marshall (Marshall AFL) was developed in the linear group (4/20). No cases of Marshall AFL were observed in the LVA group.

### Differences in AF recurrence according to ablation strategy in patients with and without LVA

**Fig. 5:**
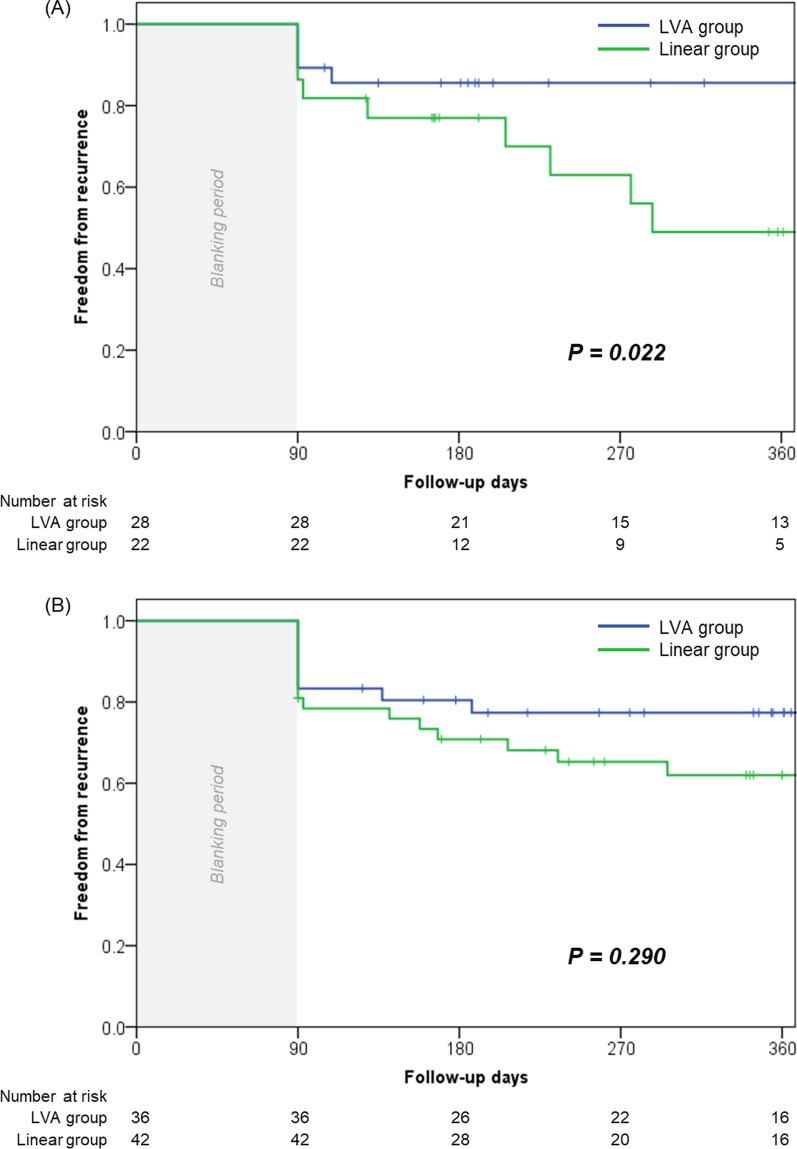
Kaplan–Meier event-free survival curves of recurrence-free rate after ablation between the linear and LVA approach groups in the patients with LVA (**A**) and without LVA (**B**). *LVA* low voltage area

Patients were divided into those with LVA (n = 50) and without LVA (n = 78), and the prognosis between the two strategies in each group was compared. Recurrence occurred less frequently in the LVA approach group than in the linear approach group among the patients with LVA (14.3% [4/28] vs. 42.9% [10/22], *p* = 0.025), while this trend was not observed in those without LVA (27.8% [10/36] vs. 40.5% [17/42], *p* = 0.340). Kaplan–Meier survival curves showed a significantly better prognosis for recurrence-free AF in the LVA approach group than in the linear approach group among patients with LVA (Fig. 5A; *p* = 0.022). In contrast, among the patients without LVA, similar prognoses were observed between the linear approach group and the LVA approach group (patients who received PVI only) (Fig. 5B; *p* = 0.290).

## Discussion

The major findings of the present study are as follows: (1) LVA ablation in addition to PVI significantly reduced the recurrence rate compared to empirical linear ablation despite the high success rate of block lines in patients with PeAF; (2) LVA ablation contributes to a reduction in procedural time and radiofrequency energy compared to linear ablation; and (3) the LVA ablation group showed a better prognosis than the linear ablation group among patients with LVA. Whereas the empirical linear ablation did not show a superior prognosis to PVI ablation alone among patients without LVA.

Although several novel mapping approaches and device techniques have been introduced to improve the outcomes of PeAF ablation, these mapping systems often require specific mapping software programs and complex calculations, which do not reflect clinical usefulness and convenience [21–24]. Linear ablation and LVA ablation techniques, which have been advocated for a long time, are commonly and universally used in daily clinical practice, and do not require a specific technique or mapping program. It is crucial to focus on the standard approach that is widely used during the ablation procedure to evaluate its efficacy with regard to outcomes. However, few studies have compared the outcomes of empirical linear and LVA-guided ablations in patients with PeAF.

### Outcomes and advantages of LVA ablation

Although the outcomes of substrate-guided AF ablation have been reported in previous studies, they mostly involved comparison of substrate modification in addition to PVI with PVI alone [10–12, 23, 25]. Yang et al. reported that a strategy of selective electrophysiological-guided atrial substrate modification (LVA ablation) in sinus rhythm after circumferential PVI was clinically more effective than the stepwise approach (roof line, MVI, and CFAE) for non-paroxysmal AF ablation in a non-randomized study [25]. They also concluded that the single procedural success rates of LVA ablation were improved (69.8% vs. 51.3%) within 24 months. Moreover, Kircher et al. recently reported that substrate modification guided by voltage mapping was associated with a significantly higher arrhythmia-free survival rate compared with a conventional approach applying linear ablation according to AF type [19]. However, their study involved approximately half of the patients with paroxysmal AF that were unlikely to require an additional modification beyond PVI. The results of our study are consistent with those in the previous studies but are unique in that we adjusted the baseline characteristics by propensity score matching analysis to reduce potential bias with focusing the patients with persistent AF only.

Tuchiya et al. evaluated the presence or absence of LA myocardial damage in an electroanatomical mapping for AF patients, and demonstrated that the LVA was not directly related to the duration of AF persistence, but was associated with an AF substrate reflecting electrical disturbance of the LA [26, 27]. In this context, it is plausible that ablation of the electrically damaged tissue associated with the arrhythmogenic substrate resulted in the suppression of AF occurrence and better outcomes after ablation. Moreover, we adapted the strict ablation approach of scar homogenization until loss of the pacing capture on the LVA, which may further prevent the development of LVA-related AFL and further recurrence in our study.

### Linear ablation and related outcomes

Compared to the previous studies evaluating outcomes of the lateral MVI line ablation and/or anterior and vein of Marshall ablation [8, 9, 20], we successfully confirmed bidirectional block of almost all linear ablations during the first session (MVI, 96.9%; roof line, 100%). This is probably due to the fact that a considerable amount of time and RF energy was used to archive to complete the block line; however, recurrence of linear lesions was proven to some extent in the second session. Despite the high success rate of linear ablations during the first session, recurrence at the linear line was comparable to that reported previously [28, 29]. It may be difficult to achieve durable linear ablations using RF ablation alone, and the achievement of linear ablation led to extended radiation exposure and procedure time, as well as an extensive amount of RF heat. To increase the success rate and durability of the MI block, vein of Marshall modification via a chemical approach has been suggested as a benefit in several reports [29–31]. However, the role and safety of ethanol infusion in the vein of Marshall in the treatment of AF remains to be determined in further investigations [32, 33].

Sawhney et al. reported that incomplete MVI block leads to high AF recurrence [34]. Although durable linear ablation may change the outcome of AF recurrence, inadequate linear ablation creates a conduction gap, such as Marshall AFL, which is well known to be proarrhythmogenic of reentrant arrhythmia [35]. Given the development of Marshall AFL in the linear group of our study, the underlying conduction gap following the MVI line ablation could increase with the risk of development of Marshall AFL thereafter. Hence, it may be better to avoid unnecessary linear ablation in cases without documented AFL during the procedure because of the possibility of an incomplete linear ablation line and an increased risk of AF and AFL recurrence.

Our study additionally demonstrated that empirical linear ablation showed no significant benefit in terms of prognosis compared to PVI ablation alone among patients without LVA. In addition, it is noteworthy that both ablation approaches did not provide an acceptable prognosis with a high recurrence rate. Specifically, in the LVA approach group, the recurrence rate tended to be higher in patients without LVA (PVI alone) than in those with LVA (PVI plus LVA ablation). Although we acknowledge the limitation of our small sample size, an alternative ablation approach (e.g., non-PV trigger ablation) may be required to improve the outcome in PeAF patients without LVA [36–38]; at the very least, additional linear ablation or PVI alone according to the assessment of the LA voltage map might not be sufficient for PeAF patients without evidence of LVA or documented AFL.

## Study limitations

This study has several limitations. First, this was a single-center, retrospective, observational study. The mean follow-up duration was relatively short because some populations dropped out in the early phase after ablation. Although ablation strategy was generally assigned alternatively in each group, for some cases, it depended on the operators’ decision of whether to use the LVA ablation or the linear ablation in a non-randomized manner. We did not have a control group with LVA who underwent PVI alone; therefore, it remains unclear whether the presence of LVA has any impact on the outcome of AF ablation. We did not evaluate CFAE, which is another possible substrate and therapeutic target during AF. Further, sub-analyses in patients without LVA may not have an adequate sample size to compare the difference in prognosis between the two strategies. Second, we could not use a high-resolution mapping system and a multi-electrode catheter, which may have affected the estimation of the amount of LVA [39]. Third, the endpoint of LVA modification remains unclear. It may be difficult to prove recurrence of LVA due to insufficient LVA modification. In this context, detailed high-density intracardiac mapping of the LVA and induction of AF via isoproterenol infusion may be useful. However, to the best of our knowledge, no trial has proven the role of LVA in recurrence after LVA modification and changes in LVA using high-resolution LVA mapping. Because of the limited performance of intermittent follow-up examinations (e.g., 12-lead electrocardiogram, 24-h Holter monitoring), rhythm assessment in our study could have caused underestimation of the asymptomatic AF recurrence [40]. AFL recurrence after the iatrogenic gaps might more likely be detected on clinical examinations owing to its persistence, which may result in a disadvantage of the prognosis in the leaner group. Finally, although we used propensity score-matching analysis to adjust the baseline characteristics between the two ablation groups, a further randomized large-scale study will be required to validate the outcomes.

## Conclusions

LVA ablation reduced the recurrence rate compared to linear ablation despite the high rate of complete block lines in PeAF patients with LVA. Unnecessary empirical linear ablation may not be recommended because of an increased risk of iatrogenic gap and recurrence.

## Supplementary Information

**Additional file 1.** Comparison of baseline characteristics and procedural data between the linear and LVA groups in total population.

## Data Availability

The data used to support this study are available from the corresponding author on reasonable request.

## Abbreviations

AF: Atrial fibrillation
AFL: Atrial flutter
CFAE: Complex fractionated atrial electrogram
CS: Coronary sinus
CTI: Cavotricuspid isthmus
LA: Left atrium
LVA: Low voltage area
MVI: Mitral valve isthmus
PeAF: Persistent atrial fibrillation
PV: Pulmonary vein
PVI: Pulmonary vein isolation
RF: Radiofrequency
