# Supplementary material for: Comparison of the empirical linear ablation and low voltage area-guided ablation in addition to pulmonary vein isolation in patients with persistent atrial fibrillation: a propensity score-matched analysis
Source: BMC Cardiovasc Disord. 2022 Jan 22;22:13. doi: 10.1186/s12872-022-02460-9 (PMC8783511; doi:10.1186/s12872-022-02460-9)
Supplement: Supplementary file 1 — Additional file 1. Comparison of baseline characteristics and procedural data between the linear and LVA groups in total population. [file 12872_2022_2460_MOESM1_ESM.docx]

| **Supplementary Table 1.** Comparison of baseline characteristics and procedural data between the linear and LVA groups in total population | | | |  |
| --- | --- | --- | --- | --- |
|  | Linear Group (n = 71) | LVA Group (n = 71) | P-value |  |
| Age (y) | 67.0 ± 10.4 | 71.0 ± 11.0 | 0.019 |  |
| Men (%) | 54 (76.1) | 53 (74.6) | 0.847 |  |
| CHADS2 score | 2.0 ± 1.3 | 2.0 ± 1.4 | 0.304 |  |
| CHA2DS2-VASc score | 3.0 ± 1.6 | 4.0 ± 1.7 | 0.132 |  |
| AF duration (months) | 4.0 (3.0–12.0) | 10.0 (3.0–25.0) | 0.207 |  |
| Long-standing persistent AF (>1y) (%) | 25 (35.2) | 31 (43.7) | 0.271 |  |
| LAD (mm) | 41.0 ± 5.2 | 41.2 ± 7.1 | 0.585 |  |
| LVEF (%) | 67.7 ± 7.2 | 66.1 ± 10.8 | 0.677 |  |
| LA volume (mL) | 147.5 ± 36.3 | 158.9 ± 48.3 | 0.290 |  |
| Antiarrhythmic drugs | 17 (23.9) | 18 (25.4) | 0.847 |  |
| Class I | 2 (2.8) | 1 (1.4) |  |  |
| Amiodarone or Bepridil | 15 (21.1) | 17 (23.9) |  |  |
| DOACs (n) | 69 (97.2) | 69 (97.2) | >0.999 |  |
| VKA (n) | 2 (2.8) | 2 (2.8) | >0.999 |  |
| Procedure results |  |  |  |  |
| PV isolation | 71 (100) | 71 (100) | >0.999 | |
| CTI block line | 71 (100) | 71 (100) | >0.999 | |
| Roof line | 71 (100) | n/a | n/a | |
| MVI line | 63 (98.6) | n/a | n/a | |
| LVA (cm^2^) | 6.9 ± 17.1 | 11.7 ± 16.2 | 0.179 | |
| Patients with LVA (%) | 26 (36.6) | 33 (46.5) | 0.307 | |
| LA area (cm^2^) | 124.6 ± 30.9 | 115.9 ± 41.6 | 0.341 | |
| LVA/LA (%) | 6.5 ± 15.2 | 10.7 ± 24.2 | 0.244 | |

LVEF = left ventricular ejection fraction, LAD = left atrial dimension, AF = atrial fibrillation, LA = left atrium, LVA = low voltage area, DOACs = direct oral anticoagulants, VKA = vitamin K antagonists, PV = pulmonary vein, CTI = cavotricuspid isthmus, MVI = mitral isthmus line. Data are presented as mean ± standard deviation, median (first and third quartiles), or number (percentage).
